# Does a transition to accountable care in Medicaid shift the modality of colorectal cancer testing?

**DOI:** 10.1186/s12913-018-3864-5

**Published:** 2019-01-21

**Authors:** Melinda M. Davis, Paul Shafer, Stephanie Renfro, Kristen Hassmiller Lich, Jackilen Shannon, Gloria D. Coronado, K. John McConnell, Stephanie B. Wheeler

**Affiliations:** 1 Department of Family Medicine, OHSU-PSU School of Public Health, and Oregon Rural Practice-based Research Network, Oregon Health & Science University, 3181 SW Sam Jackson Park Road, Mail Code L222, Portland, OR 97239 USA; 2 Department of Health Policy & Management, University of North Carolina at Chapel Hill, Chapel Hill, NC USA; 3 Center for Health Systems Effectiveness, Oregon Health & Science University, 3181 SW Sam Jackson Park Road, Portland, OR 97239 USA; 4 OHSU-PSU School of Public Health, Oregon Health & Science University, 3181 SW Sam Jackson Park Road, Portland, OR 97239 USA; 5 Center for Health Research Northwest, Kaiser Permanente, 3800 N. Interstate Avenue, Portland, OR 97227-1098 USA; 6 Department of Health Policy & Management, Lineberger Comprehensive Cancer Center, and Center for Health Promotion & Disease Prevention, University of North Carolina at Chapel Hill, Chapel Hill, NC USA

**Keywords:** Colorectal cancer, Disparities, Medicaid, Accountable care organizations

## Abstract

**Background:**

Health care reform is changing preventive services delivery. This study explored trajectories in colorectal cancer (CRC) testing over a 5-year period that included implementation of 16 Medicaid Accountable Care Organizations (ACOs, 2012) and Medicaid expansion (2014) – two provisions of the Affordable Care Act (ACA) - within the state of Oregon, USA.

**Methods:**

Retrospective analysis of Oregon’s Medicaid claims for enrollee’s eligible for CRC screening (50–64 years) spanning January 2010 through December 2014. Our analysis was conducted and refined April 2016 through June 2018. The analysis assessed the annual probability of patients receiving CRC testing and the modality used (e.g., colonoscopy, fecal testing) relative to a baseline year (2010). We hypothesized that CRC testing would increase following Medicaid ACO formation – called Coordinated Care Organizations (CCOs).

**Results:**

A total of 132,424 unique Medicaid enrollees (representing 255,192 person-years) met inclusion criteria over the 5-year study. Controlling for demographic and regional factors, the predicted probability of CRC testing was significantly higher in 2014 (+ 1.4 percentage points, *p* < 0.001) compared to the 2010 baseline but not in 2012 or 2013. Increased fecal testing using Fecal Occult Blood Tests (FOBT) or Fecal Immunochemical Tests (FIT) played a prominent role in 2014. The uptick in statewide fecal testing appears driven primarily by a subset of CCOs.

**Conclusions:**

Observed CRC testing did not immediately increase following the transition to CCOs in 2012. However increased testing in 2014, may reflect a delay in implementation of interventions to increase CRC screening and/or a strong desire by newly insured Medicaid CCO members to receive preventive care.

**Electronic supplementary material:**

The online version of this article (10.1186/s12913-018-3864-5) contains supplementary material, which is available to authorized users.

## Background

Colorectal cancer (CRC) is a leading cause of cancer death in the United States [1, 2]. CRC screening in asymptomatic, average-risk adults ages 50–75 is recommended by the United States Preventive Services Task Force (USPSTF) and can be achieved through various modalities, including colonoscopy every ten years or using high sensitivity Fecal Occult Blood Tests (FOBT) or Fecal Immunochemical Tests (FIT) annually [3, 4]. When CRC is detected at an early localized stage, the five-year survival rate is 90% compared to 13% for cancers diagnosed at advanced stages [5]. Yet, only 63% of age eligible adults in the United States are currently up-to-date with CRC screening [6]. Disparities  in CRC screening are routinely observed in rural areas and among adults with limited education, lower incomes, or Medicaid insurance [1, 7–13].

Improving CRC screening, particularly in populations experiencing disparities, could reduce CRC incidence and mortality by up to 60% [3, 14]. Multicomponent interventions – those using two or more strategies concurrently - have been shown to improve CRC screening rates [12, 15, 16]. Yet gaps remain between our knowledge of research-tested interventions to improve CRC screening and their use in routine practice [15, 17]. Moreover, simulation modeling suggests that substantial investment (more than $3.75 million) in the implementation of evidence-based interventions to increase CRC screening would produce only incremental improvements in overall CRC screening rates (+ 0.2 to + 0.5 percentage points) in populations experiencing disparities [18].

Federal and state policies to expand access to care may improve CRC screening rates. For example, a central goal of the Affordable Care Act (ACA) was to expand insurance coverage by establishing Health Insurance Marketplaces and expanding Medicaid coverage for most low-income adults with household incomes up to 138% of the federal poverty level [19]. The ACA also required that private insurance plans cover recommended preventive services without any patient cost-sharing (e.g., copayments, deductibles, or co-insurance) [20, 21] and encouraged implementation of Accountable Care Organizations (ACOs) designed to achieve the triple aim objectives of better population health and quality of care while controlling costs [22, 23].

ACOs began in Medicare as a way to deliver high-quality, coordinated care and provide financial incentives to enhance accountability [24]. Several states are also experimenting with ACO structures within their Medicaid programs [25]. Since ACOs are paid a set amount for each patient enrolled, ACO implementation is anticipated to lead to improved coordination, wiser spending, and improved quality of care by delivering the right care to the right patient at the right time. One quality indicator across many ACO initiatives is CRC screening [26, 27]. However, the interventions that ACOs pursue and how they implement them may vary drastically and have implications on program effectiveness. To date, limited research describes how transitions from fee-for-service to ACO structures impacts the completion of CRC testing or the modalities used among adults with Medicaid coverage.

Therefore, we undertook this study to examine changes in CRC testing over a 5-year period that includes implementation of 16 regional ACOs, called Coordinated Care Organizations (CCOs), in a state Medicaid program (Oregon). The objectives of this study were to: (1) analyze the annual change in the probability of patients receiving CRC testing overall and by modality used (i.e., colonoscopy, sigmoidoscopy, fecal test) relative to a baseline year (2010) and (2) explore CCO-level modality patterns in CRC testing over time. We hypothesized that CRC testing would increase annually following CCO formation.

## Methods

Our retrospective analysis explored changes in CRC testing during a 5-year period that included the transition of Medicaid enrollees into CCOs (2012–2013) and the implementation of Medicaid expansion (2014). Our analysis was conducted and refined April 2016 through June 2018. The study was approved by the Oregon Health & Science University Institutional Review Board with a waiver of informed consent (IRB #8865).

### Setting

Oregon is noted for its experimentation with Medicaid enrollment policies [28, 29]. In 2012, Oregon initiated assignment of Oregon Health Plan Members (the state’s Medicaid Program) into 16 regionally-based CCOs. CCO characteristics are described in detail elsewhere and summarized here [30–33]. CCOs are similar to ACOs in that they are responsible for providing coordinated health care services to patients in their region while controlling costs [34]. CCOs provide coverage for than 90% of Oregon’s Medicaid population; those not enrolled in a CCO receive care through the state’s Medicaid fee-for-service program for reasons related to special health needs (e.g., medically fragile children). Each CCO is governed locally by a board consisting of healthcare providers, community members, and other stakeholders. CCOs operate within a set budget based on the number of enrollees, with fixed annual percentage increases in funding [30]. As detailed in Additional file 1, Oregon’s CCOs vary by geographic region, size (range: 11,347 to 228,263 enrollees) and racial/ethnic composition (range: 6.2 to 33.3% Hispanic). CCOs include both non-profit (*n* = 9) and for-profit (*n* = 7) entities.

CCOs are accountable to the state through the tracking of multiple quality incentive measures that encompass domains ranging from preventive care to outpatient and emergency department utilization [35]. CCOs that meet improvement targets or benchmarks are eligible for annual performance bonuses from the state [30]. CRC screening has been an incentive measure since the first year of the CCO program, with reporting initiated in 2013 [35, 36].

### Data collection and analysis

#### Data source

Administrative claims and enrollment data for Medicaid enrollees were obtained from Oregon’s Health Systems Division for a five-year period from January 1, 2010 through December 31, 2014. Claims data include all healthcare encounters that generated a billing claim for enrolled members over the study period. Claims data have been widely used to understand cancer screening patterns in diverse insured populations [9, 37].

#### Identification of eligible patients

We applied the same inclusion and exclusion criteria for each calendar year to generate the analytic sample of eligible Medicaid members: aged 50 to 64 years, not dually insured by Medicare, had a valid zip code, alive for the entire study period, and continuously enrolled in a CCO (defined as enrolled for at least 11 of 12 months). We excluded individuals enrolled in Medicare since we did not have access to Medicare claims data. Consistent with prior analyses, we also excluded enrollees that had resided in more than two counties during the entire study period [13] and those with end-stage renal disease, a terminal illness that would preclude clinicians from recommending cancer screening [9, 38]. We excluded beneficiaries with a history of CRC or total colectomy to better ensure testing eligibility [9]. A total of 132,424 unique Medicaid enrollees met inclusion criteria over the study period (see Additional file 2).

#### Primary outcome measures: CRC testing and modality used

We assessed CRC testing by colonoscopy, flexible sigmoidoscopy, or FOBT/FIT to be consistent with USPSTF guidelines active during the study window [3, 39]. In the rare event of multiple CRC testing modalities billed on the same day of service, we recorded modality based on the most invasive test received (i.e., colonoscopy > flexible sigmoidoscopy > FOBT/FIT). CRC testing was identified by International Classification of Diseases, 9th Edition, Clinical Modification (ICD-9-CM), Current Procedural Terminology (CPT), or Healthcare Common Procedure Coding System (HCPCS) codes, which are summarized in Additional file 3. For FIT/FOBT, we examined non-specific codes (i.e., 82,271, 82,272, 82,273) to explore how often they occurred concurrently with a CRC screening test-specific code and discovered that usage of non-specific codes decreased in a stepped fashion from 27.5% in 2010 to 11.0% in 2014. Because this decrement may be related to coding improvement and not differences in testing behaviors, we kept non-specific procedures in the analysis. We included both screening and diagnostic billing codes for colonoscopy in our analysis, consistent with prior studies [9, 40].

#### Statistical analysis

First, we used descriptive analyses to determine the number of eligible Medicaid enrollees, their demographic characteristics, and the observed rates of CRC testing annually.

We used multivariate linear regression at the patient-level to determine the probability of CRC testing for each calendar year with reference to the baseline year (2010). Our models controlled for patient-level characteristics including age, gender, race/ethnicity (White, African-American, Hispanic, Asian/Pacific Islander, American Indian/Alaska Native, other/unknown), urbanicity (urban, rural/frontier), chronic disease risk, and use of primary care within the calendar year. We categorized urbanicity using the ZIP-code version of the Rural-Urban Commuting Areas (RUCA) taxonomy based on population density, urbanization, and daily commuting patterns: urban (50,000 or more) versus rural (2500-49,999) and frontier (< 2500) [41]. We computed Chronic Illness Disease Payment System (CDPS) indicators from claims data to adjust for chronic disease risk [42]. Use of primary care within the calendar year was determined from claims based on date of service and a standard set of CPT codes [43]. CCO fixed effects controlled for possible clustering of CRC testing outcomes at the CCO level, and calendar quarter indicators controlled for seasonality.

In addition to regressing on overall CRC testing, we also fit similar modality-specific models - i.e., where the outcome is colonoscopy, flexible sigmoidoscopy, or fecal testing. We also evaluated our selection of a linear probability model over logistic regression, a common alternative approach for binary outcome variables. Predicted probabilities of CRC testing using the linear model were found to be highly correlated with predicted probabilities from a logistic regression (Pearson’s correlation coefficient 0.961, *p* < 0.001). When concordance between approaches is high, the linear model has the advantage of more intuitive interpretation of the coefficients of the independent variables as marginal effects.

Finally, we descriptively examined the annual percentage of enrollees in each CCO who received CRC testing overall and by modality. All statistical analyses were conducted in R version 3.2.2. To protect confidentially, we de-identified CCOs when reporting data that was not publically available.

## Results

A total of 132,424 unique Medicaid enrollees met eligibility criteria, representing 236,322 person-years over the 5-year study window. The number of enrollees that met eligibility criteria varied by study year, from a low of 20,233 in 2010 to a high of 114,681 in 2014 (following Medicaid expansion in Oregon). As summarized in Tables 1, 55.4% of eligible enrollees were female; mean age was 56.3 years old. Over half (56.1%) of these enrollees resided in urban geographic areas. The majority of Medicaid enrollees visited a primary care provider during a calendar year (78.6%) and had evidence of one or more health comorbidities based on medical claims data (77.3%). Compared to the prior year, the number of eligible patients increased in 2011 (12,121; 60%) and 2014 (79,804; 229%). These increases were concurrent with state-level changes in Medicaid eligibility criteria. In 2014, the observed composition of CRC testing modalities in eligible enrollees was 55.8% colonoscopy, 43.4% FOBT/FIT, and 0.8% flexible sigmoidoscopy.

**Table 1:** Demographics of Eligible Oregon Medicaid Enrollees, 2010–2014

Characteristic Overall	*N* = 236,322
Gender	
Female	55.4
Male	44.6
Age (average)	56.3
Race/Ethnicity	
White	73.1
African-American	3.7
Hispanic	8.4
Asian/Pacific Islander	3.4
American Indian/Alaskan Native	1.9
Other/Unknown	9.5
PCP visit during 2014	78.6
PCP visit during calendar year	82.1
Geographic area	
Urban	56.1
Rural/Frontier	43.9
Health comorbidities^a^	77.3

^a^Health comorbidities identified using Chronic Disease and Disability Payment System (CDPS) risk indicators

### Marginal changes in probability of CRC testing overall and by modality

Controlling for demographic and regional factors, the probability of any CRC testing in eligible enrollees was significantly higher than the baseline year (2010) in 2011 (0.7 percentage points, *p* < 0.001) and 2014 (1.4 percentage points, *p* < 0.001), see Table 2. While colonoscopy contributed to the increase in both 2011 and 2014 (0.5 and 0.6 percentage points, respectively), FOBT/FIT also contributed: 0.2 percentage point increase in 2011 and 0.7 percentage point increase in 2014.

**Table 2:** Marginal Annual Effects of Colorectal Cancer Testing Overall and by Modality in 16 Medicaid ACOs, 2011–2014 Compared to 2010, percentage point (*p*-value)

		CRC Screening Modality		
Year effect relative to 2010	Any CRC screening	Colonoscopy	FOBT/FIT	Flexible sigmoidoscopy
2011	0.007 (< 0.001)	0.005 (< 0.001)	0.002 (< 0.001)	< 0.001 (< 0.001)
2012	0.001 (0.333)	0.001 (0.022)	0.000 (0.691)	< 0.001 (< 0.001)
2013	0.001 (0.104)	−0.001 (0.186)	0.002 (< 0.001)	−0.001 (< 0.001)
2014	0.014 (< 0.001)	0.006 (< 0.001)	0.007 (< 0.001)	< 0.001 (< 0.001)

Note: Each column represents a separate regression model. These coefficients represent the annual marginal change in the predicted probability of the outcome relative to the baseline year (2010). Each model also controls for age, gender, race/ethnicity, chronic disease risk, urbanicity, PCP visit during the calendar year, CCO assignment, and calendar quarter. Results are interpreted as percentage point changes compared to a 2010 baseline

### Observed levels of CRC testing and modality patterns by CCO

Observed levels of CRC testing statewide and by CCO are presented in Additional file 4. Statewide, the observed level of annual CRC testing was 3.2 percentage points higher in 2014 compared to 2010 (16.2% versus 13.0%). By individual CCO, the relative difference for any evidence of annual CRC testing in 2014 compared to 2010 ranged from a decrease of 0.5 percentage points (− 0.5, CCO N) to an increase of 9.8 percentage points (+ 9.8, CCO E).

**Fig. 1:**
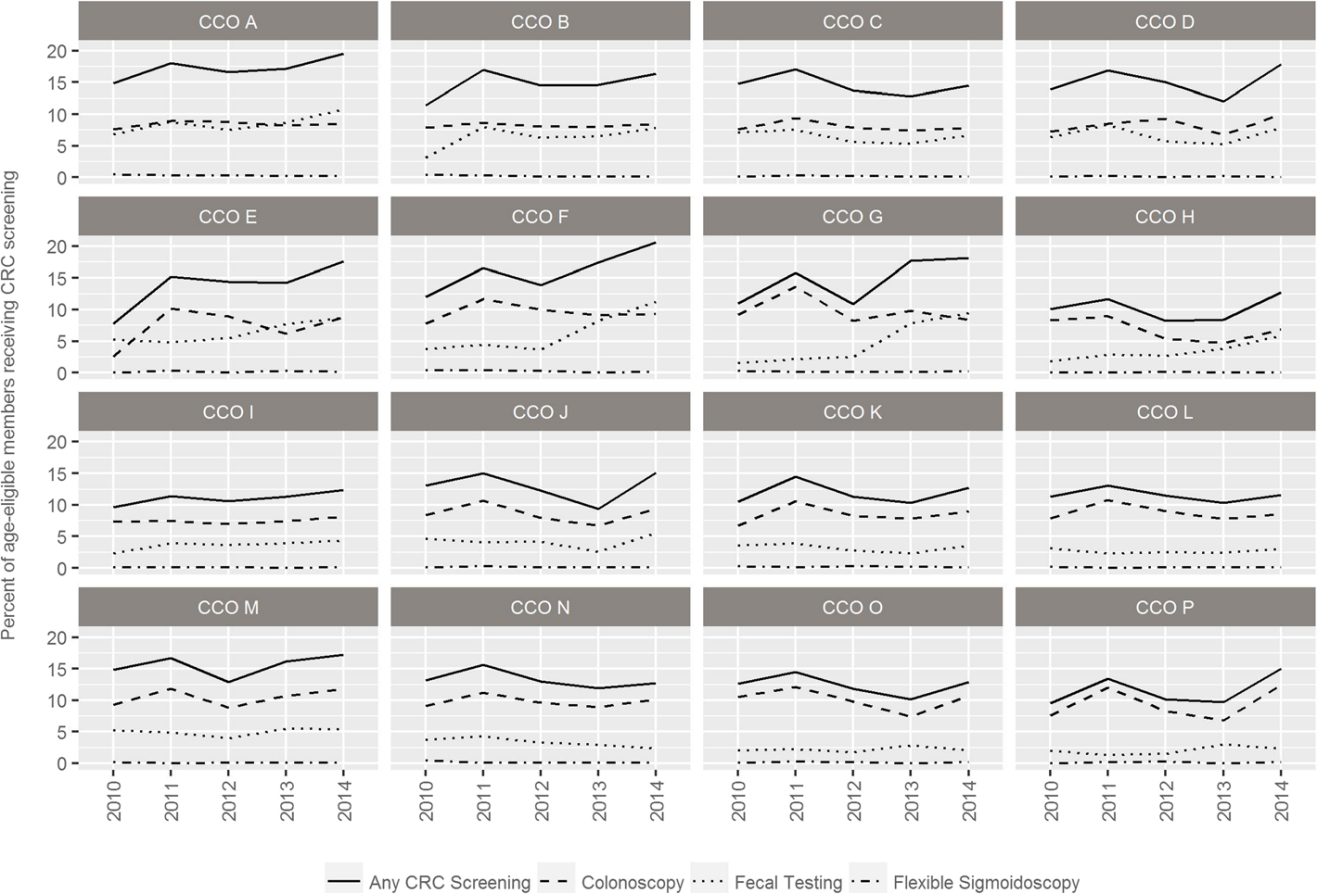
Annual Observed CRC Testing Trajectories and Modality Used in Eligible Enrollees in 16 Medicaid ACOs, 2010–2014

CRC testing modality patterns varied within and across CCOs over time. Figure 1 illustrates trajectories for CRC testing annually overall and by modality type for each CCO. Use of flexible sigmoidoscopy was rare across all CCOs. Some CCOs displayed a relatively high level of CRC testing using FOBT/FIT across all study years (i.e., CCOs A - E), whereas others demonstrated a relatively low level of FOBT/FIT use (i.e., CCOs I – P). Two CCOs (F and G) displayed distinct upticks in CRC testing using FOBT/FIT following formation of CCOs in 2012. Many CCOs displayed a spike in CRC testing in 2011, driven by colonoscopy usage, and a few CCOs displayed an uptick in overall CRC testing in 2014 that appeared to be driven by colonoscopy (i.e., CCOs 0 and P) or by a combination of colonoscopy and FOBT/FIT (i.e., CCOs D - J).

## Discussion

This study explored changes in CRC testing and the modality used among Medicaid enrollees in Oregon over a five-year period that included implementation of numerous ACA provisions, such as the transition of Medicaid members into 16 ACOs (CCOs, 2012) and Medicaid expansion (2014). Compared to the baseline year (2010) the probability of CRC testing statewide significantly increased in 2011 and 2014 by 0.7 and 1.4 percentage points, respectively. Modality of CRC testing varied by CCO over time related to consistently high or low use of FIT/FOBT or an uptick in FIT/FOBT following CCO formation.

This increase in annual CRC testing in 2011 and 2014 statewide may be driven in part by the increased number of eligible enrollees in both years (12,121 or 60% in 2011; 79,804 or 229% in 2014) – which corresponds with changes in state-level eligibility requirements for Medicaid enrollment. This adds to the robust body of research that demonstrates that insurance coverage is an important predictor of preventive screening [28, 29]. However, it is notable that the modality driving these CRC testing upticks varied. While colonoscopy drove the 2011 uptick, FOBT/FIT played a prominent role in the 2014 increase. The uptick in 2014 may be related to the formation of CCOs and the subsequent interventions they implemented to increase CRC testing – one CCO quality incentive metric. Variation in modality patterns within individual CCOs suggests that these health plans may implement different interventions or implement the same interventions in more or less effective ways [44].

The ACA has encouraged rapid transformation of health systems with the triple of aim of improving care quality and patient outcomes while controlling costs [22]. Evidence describing how ACO payment structures impact utilization of preventive services and clinical outcomes in Medicaid patients is still emerging [32]. This is the first study, to our knowledge, to examine changes in CRC testing in a context that includes formation of Medicaid ACOs and implementation of other ACA provisions. Although ACOs originated in Medicare, by 2018 12 states had implemented some form of ACO program within Medicaid, and 10 more were pursing them [25]. The effectiveness of ACO structures on preventive care delivery, which includes CRC screening, appears dependent on organization characteristics [45–47]. Building relationships, providing actionable data, and supporting quality improvement infrastructure are critical dimensions when CCOs work with primary care clinics to implement interventions to increase CRC screening [44]. Uncertainty about the future of the ACA underscores the need for research like ours to understand how such health reforms affect patients’ receipt of preventive services, such as CRC screening.

Policies provide standards and guidance for ACOs, health systems, and individual primary care practices. However, decisions on what and how to implement interventions to achieve policy targets are often determined based on local context and stakeholder priorities. Interventions to increase CRC screening and address cancer disparities may benefit by focusing on local contexts as the starting point to inform intervention selection, alignment, and implementation [17, 48, 49]. Research exploring how to help ACOs select and implement interventions that are evidence based and best suited to their specific patient populations, clinical settings, and community characteristics [15] by modeling intervention impact [18] or using participatory implementation science methods [49] is needed. Future studies could also explore how the interventions implemented within ACOs interact with patient-level characteristics to explain variation in modality of CRC testing over time. For example, additional research is needed to determine if patients that are newly insured following Medicaid expansion are highly receptive to completing preventive screenings such as CRC testing in order to “catch up” on their health care.

Although the percentage of individuals who are up-to-date with CRC screening is improving nationally, further movement is needed to achieve the National Colorectal Cancer Roundtable (NCCRT) target of 80% and to address disparities in vulnerable populations (e.g., minorities, Medicaid enrollees, rural patients) [1, 7, 12, 50–52]. Many evidence-based interventions increase CRC screening and a number of federally sponsored initiatives such NCI’s Cancer Moonshot and the Cancer Prevention and Control Research Network (CPCRN) [53] are designed to speed translation of this evidence into routine practice. Achieving national targets for CRC screening would prevent 277,000 new cases of colon cancer and 203,000 deaths within 20 years [54]. Understanding how policies and interventions are being effectively implemented within states and individual Medicaid ACOs may contribute to increased CRC screening and ultimately help eliminate observed disparities.

Our study has several limitations. First, we relied on administrative claims to quantify the percentage of eligible enrollees that were tested for CRC in any calendar year rather than the proportion of enrollees who were up-to-date. We chose this approach because we lacked access to claims data needed to monitor colonoscopy completion. This approach likely yields a greater frequency of FOBT/FIT testing compared to colonoscopy because of the disparate recommended screening windows (annually versus every 10 years). Second, we were unable to compare changes in CRC screening in our Medicaid population to a control group because most of the state’s Medicaid population was enrolled in CCOs and only a small, remarkably dissimilar population remained in fee for service. Our findings over time, and within CCOs, suggest that research comparing observed changes in CRC testing in ACO programs and sites not implementing ACO structures is warranted. Finally, since we focused on Medicaid ACOs in Oregon, it is unknown if observed patterns would appear during ACO implementation among non-Medicaid populations (e.g., commercially insured, uninsured) or in different geographic regions. Despite these limitations, our study provides preliminary data to shape future research on the impact of ACA provisions and ACO implementation on CRC screening and may apply to other preventive services.

## Conclusion

Our study suggests that ACO formation and Medicaid expansion – two provisions of the ACA – are associated with increased CRC testing. Compared to the 2010 baseline, the probability of CRC testing in Medicaid enrollees increased by 1.4 percentage points in 2014. This delay in increased CRC testing following the transition to CCOs in 2012, may reflect the time required for implementation of interventions to increase CRC screening and/or a strong desire by newly insured Medicaid CCO members to receive preventive care. While we observed that increased CRC testing was associated with years in which there was a greater number of Medicaid enrollees (2011, 2014) – it is notable that the 2014 increase was also driven by an increase in the use of FIT/FOBT compared to prior years. These findings suggest that formation of CCOs, with an incentive metric related to CRC screening, contributed to increased CRC testing driven in part by greater use of FIT/FOBT. Given that simulation modeling suggests that investments of $3.75 million to support implementation of evidence-based interventions would yield only incremental improvements in CRC testing (+ 0.2 to + 0.5 percentage points) in underserved populations [18]; the observed 1.4 percentage point increase in 2014 appears to be substantial. Further study of the relationships between the interventions used, implementation strategies, and improvements in testing across the CCOs, and in other ACO and non-ACO settings, is warranted.

## Additional files

Additional file 1: Characteristics of Oregon’s 16 Coordinated Care Organizations (CCOs), 2014 Public Data**.** This table provides a snapshot of Oregon’s 16 CCOs by organizational structure, nonprofit status, location, size (number of enrollees) and the percent of eligible Medicaid members who were up-to-date for CRC screening based on 2014 public data. (DOCX 14 kb)

Additional File 2: Inclusion and exclusion criteria applied to generate analytic sample for Medicaid members**.** This table summarizes where individuals “fell out” of the analytic sample as we applied our inclusion and exclusion criteria. The lines in the table parallel the descriptors that are presented in the manuscript text. (DOCX 12 kb)

Additional File 3: Billing codes indicating colorectal cancer screening procedures or exclusion criteria**.** This table presents all of the CPT, HCPCS, and ICD-9 Procedure codes that we applied to our claims data in order to assess the primary outcome of CRC testing and modality used. We present our codes for screening test modality and exclusion reason. This information is provided to support transparency and help others who are doing this work. Because our analysis was on data collected prior to 2014, we did not present ICD-10 Procedure codes in this table but is available from the authors upon request. (DOCX 13 kb)

Additional File 4: Observed Levels of Any Colorectal Cancer Testing State-wide and by Medicaid ACO, 2010 to 2014**.** This table summarizes observed levels of CRC testing across all five study years by individual CCO, and across the state. This information complements the data presented in Fig. 1 and is provided in case readers would like to see the actual numeric value displayed for individual CCOs. (DOCX 13 kb)

## Abbreviations

ACA: Affordable Care Act
ACO: Accountable Care Organization
CCO: Coordinated Care Organization
CPT: Current Procedure Terminology
CRC: Colorectal Cancer
FIT: Fecal Immunochemical Test
FOBT: Fecal Occult Blood Test
HCPCS: Healthcare Common Procedure Coding System
ICD-9-CM: International Classification of Diseases, 9th Edition, Clinical Modification
USPSTF: United States Preventive Services Task Force

## References

[1] White A, Thompson TD, White MC (2017). Cancer screening test use — United States, 2015. MMWR.

[2] American Cancer Society. Cancer Statistics Center. 2018; https://cancerstatisticscenter.cancer.org/#!/. Accessed 7 Jan 2019.

[3] Whitlock EP, Lin JS, Liles E, Beil TL, Fu R (2008). Screening for colorectal Cancer: a targeted, updated systematic review for the U.S. preventive services task Force. Ann Intern Med.

[4] Force USPST, Bibbins-Domingo K, Grossman DC (2016). Screening for colorectal Cancer: US preventive services task Force recommendation statement. JAMA.

[5] American Cancer Society. Colorectal Cancer Facts & Figures 2014-2016. Atlanta: American Cancer Society. 2014.

[6] American Cancer Society. Colorectal Cancer Facts & Figures 2017-2019. Atlanta: American Cancer Society. 2017.

[7] Cole AM, Jackson JE, Doescher M (2012). Urban-rural disparities in colorectal cancer screening: cross-sectional analysis of 1998-2005 data from the centers for disease Control's behavioral risk factor surveillance study. Cancer medicine.

[8] Morbidity, and Mortality Weekly Report (MMWR). Vital Signs (2013). Colorectal Cancer screening test use - United States, 2012. MMWR.

[9] Wheeler SB, Kuo TM, Goyal RK (2014). Regional variation in colorectal cancer testing and geographic availability of care in a publicly insured population. Health & place.

[10] Ojinnaka CO, Choi Y, Kum HC, Bolin JN (2015). Predictors of colorectal Cancer screening: does rurality play a role?. J Rural Health.

[11] Anderson AE, Henry KA, Samadder NJ, Merrill RM, Kinney AY (2013). Rural vs urban residence affects risk-appropriate colorectal cancer screening. Clin Gastroenterol Hepatol.

[12] Holden DJ, Jonas DE, Porterfield DS, Reuland D, Harris R (2010). Systematic review: enhancing the use and quality of colorectal cancer screening. Ann Intern Med.

[13] Davis M, Renfro S, Pham R (2017). Geographic and population-level disparities in colorectal Cancer testing: a multilevel analysis of Medicaid and commercial claims data. Prev Med.

[14] Vogelaar I, van Ballegooijen M, Schrag D (2006). How much can current interventions reduce colorectal cancer mortality in the U.S.? Mortality projections for scenarios of risk-factor modification, screening, and treatment. Cancer.

[15] Davis M, Freeman M, Shannon J, et al. A systematic review of clinic and community intervention to increase fecal testing for colorectal Cancer in rural and low-income populations in the United States – how, what and when? BMC Cancer. 2018;18(40). 10.1186/s12885-017-3813-4 PMC5756384 29304835

[16] The Community Guide. Cancer screening: multicomponent interventions - colorectal Cancer. 2016; https://www.thecommunityguide.org/findings/cancer-screening-multicomponent-interventions-colorectal-cancer. Accessed 7 Jan 2019.

[17] Wheeler SB, Davis MM (2017). “Taking the bull by the horns”: four principles to align public health, primary care, and community efforts to improve rural Cancer control. J Rural Health.

[18] Hassmiller Lich K, Cornejo DA, Mayorga ME (2017). Cost-effectiveness analysis of four simulated colorectal Cancer screening interventions, North Carolina. Prev Chronic Dis.

[19] Henry J. Kaiser Family Foundation. Status of state action on the Medicaid expansion decision. 2019. https://www.kff.org/health-reform/state-indicator/state-activity-around-expanding-medicaid-under-the-affordable-care-act/?currentTimeframe=0&sortModel=%7B%22colId%22:%22Location%22,%22sort%22:%22asc%22%7D. Accessed 7 Jan 2019.

[20] Patient Protection and Affordable Care Act H.R. 3590,,, (2006).

[21] The Henry J. Kaiser Family Foundation. Coverage of colonoscopies under the Affordable Care Act's prevention benefit. September 2012.

[22] Berwick DM, Nolan TW, Whittington J (2008). The triple aim: care, health, and cost. Health Aff.

[23] Herrel L, Norton E, Hawken S, Ye Z, Hollenbeck B, Miller D (2016). Early impact of Medicare accountable care organizations on cancer surgery outcomes. Cancer.

[24] Centers for Medicare & Medicaid Services. Accountable Care Organizations (ACOs). 2018; https://www.cms.gov/Medicare/Medicare-Fee-for-Service-Payment/ACO/. Accessed 7 Jan 2019.

[25] Center for Health Care Strategies Inc. Medicaid accountable care organizations: State update. 2018. https://www.chcs.org/resource/medicaid-accountable-care-organizations-state-update/. Accessed 7 Jan 2019.

[26] Kaiser Family Foundation. Mapping Medicaid Delivery System and Payment Reform. 2017. http://kff.org/interactive/delivery-system-and-payment-reform/. Accessed 7 Jan 2019.

[27] Wang H, Qiu F, Gregg A (2018). Barriers and facilitators of colorectal Cancer screening for patients of rural accountable care organization clinics: a multilevel analysis. J Rural Health..

[28] Marino M, Bailey SR, Gold R (2016). Receipt of preventive services after Oregon’s randomized Medicaid experiment. Am J Prev Med.

[29] Allen H, Baicker K, Finkelstein A, Taubman S, Wright BJ (2010). The Oregon health study G. what The Oregon health study can tell us about expanding Medicaid. Health affairs (Project Hope).

[30] Stock R, Goldberg B, eds. *Health Reform Policy to Practice: Oregon's Path to a Sustainable Health System, A Study in Innovation.* Academic Press; 2017.

[31] McConnell KJ (2016). Oregon’s Medicaid coordinated care organizations. JAMA.

[32] McConnell KJ, Renfro S, Chan BK, et al. Early performance in Medicaid accountable care organizations: a comparison of Oregon and Colorado. JAMA Intern Med. 2017. 10.1001/jamainternmed.2016.9098 PMC5440252 28192568

[33] McConnell KJ, Renfro S, Lindrooth RC, Cohen DJ, Wallace NT, Chernew ME (2017). Oregon’s Medicaid reform and transition to global budgets were associated with reductions in expenditures. Health Aff.

[34] McConnell KJ, Chang AM, Cohen DJ (2014). Oregon’s Medicaid transformation: an innovative approach to holding a health system accountable for spending growth. Healthcare (Amsterdam, Netherlands).

[35] Oregon Health Authority. CCO Incentive Measures since 2013. 2016.

[36] Oregon Health Authority. Oregon's Health System Transformation: CCO Metrics 2015 Final Report. 2016.

[37] Gupta S, Tong L, Anderson P (2013). Measurement of colorectal cancer test use with medical claims data in a safety-net health system. Am J Med Sci.

[38] Wheeler SB, Carpenter WR, Peppercorn J, Schenck AP, Weinberger M, Biddle AK (2012). Structural/organizational characteristics of health services partly explain racial variation in timeliness of radiation therapy among elderly breast cancer patients. Breast Cancer Res Treat.

[39] Force USPST (2016). Screening for colorectal cancer: us preventive services task force recommendation statement. JAMA.

[40] Schenck AP, Klabunde CN, Warren JL (2007). Data sources for measuring colorectal endoscopy use among Medicare enrollees. Cancer Epidemiology Biomarkers &amp; Prevention.

[41] Rural-Urban Commuting Area Codes. 2005 http://depts.washington.edu/uwruca/ruca-codes.php. Accessed 7 Jan 2019.

[42] Kronick R, Gilmer T, Dreyfus T, Lee L (2000). Improving health-based payment for Medicaid beneficiaries: CDPS. Health care financing review.

[43] Chang C, Stukel TA, Flood A, Goodman DC (2011). Primary care physician workforce and medicare beneficiaries' health outcomes. JAMA.

[44] Davis MM, Gunn R, Pham R, et al. How do Accountable Care Organizations work with Primary Care Clinics to Improve Colorectal Cancer Screening? Relationships, Data, and Improvement Strategies. In Review. 10.5888/pcd16.180395 PMC6716418 31418685

[45] Ticse C (2016). Certain organizational characteristics affect ACO preventive care quality performance. Findings brief : health care financing & organization.

[46] Albright BB, Lewis VA, Ross JS, Colla CH (2016). Preventive care quality of Medicare accountable care organizations: associations of organizational characteristics with performance. Med Care.

[47] Wharam JF, Zhang F, Landon BE, LeCates R, Soumerai S, Ross-Degnan D (2016). Colorectal Cancer screening in a Nationwide high-deductible health plan before and after the affordable care act. Med Care.

[48] Wheeler SB, Basch E (2017). Translating Cancer surveillance data into effective public health interventions. JAMA.

[49] Ramanadhan S, Davis MM, Armstrong R (2018). Participatory implementation science to increase the impact of evidence-based cancer prevention and control. Cancer Causes Control.

[50] Mokdad AH, Dwyer-Lindgren L, Fitzmaurice C (2017). Trends and patterns of disparities in cancer mortality among us counties, 1980-2014. JAMA.

[51] Henley SJ, Anderson RN, Thomas CC, Massetti GM, Peaker B, LC R. Invasive Cancer incidence, 2004–2013, and deaths, 2006–2015, in nonmetropolitan and metropolitan counties — United States. MMWR Surveill Summ 2017;66(SS-14):1–13. 10.15585/mmwr.ss6614a1 PMC5879727 28683054

[52] Fedewa SA, Corley DA, Jensen CD, et al. Colorectal Cancer screening initiation after age 50 years in an organized program. Am J Prev Med. 53(3):335–44. 10.1016/j.amepre.2017.02.018 PMC5562515 28427954

[53] Fernandez ME, Melvin CL, Leeman J (2014). The cancer prevention and control research network: an interactive systems approach to advancing cancer control implementation research and practice. Cancer Epidemiol Biomarkers Prev.

[54] Simon S. Achieving 80% by 2018 screening goal could prevention 200,000 colon cancer deaths in less than 2 decades. 2015. https://www.cancer.org/latest-news/impact-of-achieving-80-by-2018-screening-goal.html. Accessed 7 Jan 2019.

