# Supplementary material for: Does a transition to accountable care in Medicaid shift the modality of colorectal cancer testing?
Source: BMC Health Serv Res. 2019 Jan 21;19:54. doi: 10.1186/s12913-018-3864-5 (PMC6341697; doi:10.1186/s12913-018-3864-5)
Supplement: Supplementary file 1 — Characteristics of Oregon’s 16 Coordinated Care Organizations (CCOs), 2014 Public Data. This table provides a snapshot of Oregon’s 16 CCOs by organizational structure, nonprofit status, location, size (number of enrollees) and the percent of eligible Medicaid members who were up-to-date for CRC screening based on 2014 public data. (DOCX 14 kb) [file 12913_2018_3864_MOESM1_ESM.docx]

**Additional File 1. Characteristics of Oregon’s 16 Coordinated Care Organizations (CCOs), 2014 Public Data**

| CCO Name | Structure | Nonprofit status | Location | Number of enrollees | % Up-to-Date for CRC Screening |
| --- | --- | --- | --- | --- | --- |
| AllCare Health Plan | corporation | no | Urban | 47,178 | 29.7 |
| Cascade Health Alliance | LLC | no | Rural | 15,636 | 54.0 |
| Columbia Pacific | LLC | yes | Rural | 25,530 | 31.6 |
| Eastern Oregon | LLC | no | Rural | 44,801 | 35.3 |
| FamilyCare | corporation | yes | Urban | 110,324 | 47.4 |
| Health Share of Oregon | corporation | yes | Urban | 225,068 | 53.3 |
| Intercommunity Health Network | corporation | yes | Rural | 52,742 | 51.8 |
| Jackson Care Connect | LLC | yes | Urban | 27,828 | 47.0 |
| PacificSource–Central Oregon | corporation | yes | Urban | 50,875 | 53.5 |
| PacificSource–Gorge | corporation | yes | Urban | 12,244 | 46.7 |
| PrimaryHealth of Josephine County | LLC | yes | Rural | 10,565 | 40.5 |
| Trillium | corporation | no | Urban | 72,187 | 50.1 |
| Umpqua Health Alliance | LLC | no | Rural | 25,195 | 51.7 |
| Advanced Health | LLC | no | Rural | 19,614 | 52.1 |
| Willamette Valley Community Health | LLC | no | Urban | 93,357 | 48.4 |
| Yamhill | corporation | yes | Rural | 20,753 | 46.7 |

From publically available data from Oregon Secretary of State filings, Oregon Health Authority reports, and CCO websites. 2014 up-to-date status was determined by the state using claims data and chart audits for a random sample of patients from each CCO.
