# Supplementary material for: Does a transition to accountable care in Medicaid shift the modality of colorectal cancer testing?
Source: BMC Health Serv Res. 2019 Jan 21;19:54. doi: 10.1186/s12913-018-3864-5 (PMC6341697; doi:10.1186/s12913-018-3864-5)
Supplement: Supplementary file 2 — Inclusion and exclusion criteria applied to generate analytic sample for Medicaid members. This table summarizes where individuals “fell out” of the analytic sample as we applied our inclusion and exclusion criteria. The lines in the table parallel the descriptors that are presented in the manuscript text. (DOCX 12 kb) [file 12913_2018_3864_MOESM2_ESM.docx]

**Additional File 2. Inclusion and exclusion criteria applied to generate analytic sample for Medicaid members**

| **Criteria** | **Medicaid N (Overall)** |
| --- | --- |
| Total unique members | 1,564,618 |
| Age 50-64 | 216,504 |
| Drop Medicaid/Medicare dual eligibles | 173,563 |
| Keep members enrolled in a CCO | 169,832 |
| Continuously enrolled 11/12 months during calendar year | 136,904 |
| Alive at end of study period | 133,394 |
| Resident of no more than two counties | 133,254 |
| No history of CRC/total colectomy | 132,830 |
| No history of ESRD | 132,424 |
