# Supplementary material for: Does a transition to accountable care in Medicaid shift the modality of colorectal cancer testing?
Source: BMC Health Serv Res. 2019 Jan 21;19:54. doi: 10.1186/s12913-018-3864-5 (PMC6341697; doi:10.1186/s12913-018-3864-5)
Supplement: Supplementary file 3 — Billing codes indicating colorectal cancer screening procedures or exclusion criteria. This table presents all of the CPT, HCPCS, and ICD-9 Procedure codes that we applied to our claims data in order to assess the primary outcome of CRC testing and modality used. We present our codes for screening test modality and exclusion reason. This information is provided to support transparency and help others who are doing this work. Because our analysis was on data collected prior to 2014, we did not present ICD-10 Procedure codes in this table but is available from the authors upon request. (DOCX 13 kb) [file 12913_2018_3864_MOESM3_ESM.docx]

**Additional File 3. Billing codes indicating colorectal cancer screening procedures or exclusion criteria**

| **Category** | **Codes** |
| --- | --- |
| **Screening test modality** |  |
| Fecal occult blood test (FOBT) or Fecal Immunochemical Test (FIT) | CPT: 82270 (FOBT), 82274 (FIT); 82272*, 82273*, 82271*  HCPCS: G0328 (FIT), G0107 (FOBT) |
| Colonoscopy | CPT: 44388, 44389, 44390, 44391, 44392, 44393, 44394, 44397, 45355, 45378, 45379, 45380, 45381, 45382, 45383, 45384, 45385, 45386, 45387, 45391, 45392  HCPCS: G0105, G0121  ICD-9 Procedure: 45.21, 45.22, 45.23, 45.25, 45.41, 45.42, 45.43, 48.36 |
| Flexible Sigmoidoscopy | CPT: 45300, 45303, 45305, 45307, 45308, 45309, 45315, 45317, 45320, 45321, 45327, 45330, 45331, 45332, 45333, 45334, 45335, 45337, 45338, 45339, 45340, 45341, 45342, 45345  HCPCS: G0104  ICD-9 Procedure: 45.24, 48.21, 48.22, 48.23, 48.24 |
| **Exclusion Reason** |  |
| Colorectal Cancer | HCPCS: G0213, G0214, G0215, G0231  ICD-9 Diagnosis: 153, 154.0, 154.1, 154.2, 154.3, 197.5, V10.05, V10.06 |
| Total Colectomy | CPT: 44150, 44151, 44152, 44153, 44155, 44156, 44157, 44158, 44210, 44211, 44212  ICD-9 Procedure: 45.8, 45.81, 45.82, 45.83 |
| End Stage Renal Disease | ICD-9 Diagnosis: 585.6 |

Abbreviations: ICD-9-CM = International Classification of Diseases, 9^th^ Edition, Clinical Modification (ICD-9-CM), CPT = Current Procedural Terminology, HCPCHS = Healthcare Common Procedure Coding System

* Indicates a non-specific code for fecal testing; 82271 replaced 82273 in 2006.

Note: We explored billing codes for Barium enema, CT colonography, fecal DNA, and CRC screening review/documentation in descriptive statistics. These codes were infrequently used in claims and they were excluded from the final analysis because they were not endorsed modalities for CRC screening by current national guidelines.
