# Supplementary material for: Does a transition to accountable care in Medicaid shift the modality of colorectal cancer testing?
Source: BMC Health Serv Res. 2019 Jan 21;19:54. doi: 10.1186/s12913-018-3864-5 (PMC6341697; doi:10.1186/s12913-018-3864-5)
Supplement: Supplementary file 4 — Observed Levels of Any Colorectal Cancer Testing State-wide and by Medicaid ACO, 2010 to 2014. This table summarizes observed levels of CRC testing across all five study years by individual CCO, and across the state. This information complements the data presented in Fig. 1 and is provided in case readers would like to see the actual numeric value displayed for individual CCOs. (DOCX 13 kb) [file 12913_2018_3864_MOESM4_ESM.docx]

**Additional File 4. Observed Levels of Any Colorectal Cancer Testing State-wide and by Medicaid ACO, 2010 to 2014**

|  | **Study Year** | | | | |  |
| --- | --- | --- | --- | --- | --- | --- |
| CCO Identifier | 2010  (N = 20,233) | 2011  (N = 32,354) | 2012  (N = 34,177) | 2013  (N = 34,877) | 2014  (N = 114,681) | Relative Difference, 2014 - 2010 |
| A | 14.9 | 18.0 | 16.6 | 17.2 | 19.5 | +4.6 |
| B | 11.4 | 17.0 | 14.5 | 14.6 | 16.4 | +5.0 |
| C | 14.8 | 17.1 | 13.7 | 12.8 | 14.5 | -0.3 |
| D | 13.9 | 16.9 | 15.0 | 12.0 | 17.9 | +4.0 |
| E | 7.8 | 15.2 | 14.4 | 14.2 | 17.6 | +9.8 |
| F | 12.0 | 16.5 | 13.9 | 17.4 | 20.6 | +8.6 |
| G | 11.0 | 15.9 | 10.9 | 17.7 | 18.1 | +7.1 |
| H | 10.1 | 11.7 | 8.2 | 8.4 | 12.7 | +2.6 |
| I | 9.6 | 11.4 | 10.6 | 11.3 | 12.4 | +2.8 |
| J | 13.1 | 15.0 | 12.3 | 9.4 | 15.0 | +1.9 |
| K | 10.5 | 14.6 | 11.3 | 10.3 | 12.7 | +2.2 |
| L | 11.3 | 13.1 | 11.5 | 10.3 | 11.6 | +0.3 |
| M | 14.8 | 16.6 | 12.9 | 16.2 | 17.2 | +2.4 |
| N | 13.2 | 15.6 | 13.0 | 11.9 | 12.7 | -0.5 |
| O | 12.8 | 14.5 | 11.8 | 10.2 | 12.9 | +0.1 |
| P | 9.5 | 13.4 | 10.2 | 9.7 | 15.0 | +5.5 |
| Oregon Statewide | 13.0 | 15.9 | 13.7 | 13.9 | 16.2 | +3.2 |
